# Magnitude and associated factors of undernutrition (underweight and stunting) among school adolescent girls in Hawzen Woreda (District), Tigray regional state, Northern Ethiopia: Cross-sectional study

**DOI:** 10.1186/s13104-020-4926-4

**Published:** 2020-02-07

**Authors:** Kidanemaryam Berhe, Gebrehiwot Gebremariam

**Affiliations:** 1 Department of Nutrition and Dietetics, School of Public Health, College of Health Sciences, Mekelle University, Tigray, Ethiopia; 2 Public Health Department, College of Health Sciences, Adigrat University, Tigray, Ethiopia

**Keywords:** Underweight, Stunting, Adolescent girls, Hawzen

## Abstract

**Objective:**

There is a lack of evidence concerning undernutrition (underweight and stunting) among adolescent girls in the study area, therefore, this study aimed to assess the magnitude of undernutrition and associated factors among adolescent girls in Hawzen woreda, Northern Ethiopia.

**Results:**

This study found that 32.2% and 33.2% of school adolescent girls were underweight and stunted respectively. Illiterate fathers were higher among underweight adolescent girls as compared to those normal-weight adolescent girls (AOR = 1.4, 95% CI; 1.1, 1.7). Underweight adolescents were higher among low-grade level adolescents (grade 4–8) as compared to grade 9–12 adolescents (AOR = 2, 95% CI; 1.4, 2.6). Unemployed mothers were higher among stunted adolescent girls as compared to the counterpart (AOR = 2.2, 95% CI; 1.1, 3.3). It would be good to consider the above-mentioned factors during the nutritional intervention of adolescents.

## Introduction

World Health Organization (WHO) defined adolescents as persons aged 10–19 years [1]. Adolescents gain fifty percent of adult weight and more than twenty percent of their adult height during this period. The adolescents are generally expected to enjoy good health and being less vulnerable to infection compared with under-five children and of chronic diseases compared with aging people, and hence they have generally being given little health and nutritional attention except for reproductive health concern. Undernutrition is a major public health problem among adolescent girls all over the world. In developing countries including Ethiopia, half of all adolescents fail to achieve their full genetic growth potential. Poor dietary diversity, poor dietary inadequacies, frequent illness, lack of health care access, increased nutritional requirement can affect adolescent nutrition [2–4]. Undernourished adolescents have lowered resistance to infection; they are more likely vulnerable to common diseases like diarrheal diseases and respiratory infections [5–8]. Adolescent’s undernutrition in Asia and Africa is generally higher with a magnitude of 32–65% and 4–30% respectively. In Sub Sahara Africa, the magnitude of adolescence under-nutrition is 15–58%, which is higher from other African countries [6, 9, 10]. There is a lack of evidence concerning adolescent girls’ undernutrition in the study area. Therefore, this study aimed to assess the magnitude and associated factors of undernutrition (underweight and stunting) among adolescent girls in Hawzen woreda, Northern Ethiopia.

## Main text

### Methods and materials

The study was conducted in Hawzen woreda, Tigray, Northern Ethiopia at school level from December 2017 to January 2018. Hawzen woreda is located in the Eastern part of the Tigray regional state, at a distance of 950 km from Addis Ababa and 84 km from Mekelle town. According to the 2007 Central Statistics Agency of Ethiopia (CSA), the woreda has a total population of 117,954. The woreda has 34 schools; 30 of them are primary (1–8 grade) and 4 are secondary (9–12 grade) schools. A total of 29,840 students (15,568 girls) attend their education in these schools.

The institution (school) based cross-sectional study design was applied and the study population was adolescent girl students attending their education in selected primary and secondary schools of Hawzen woreda. Adolescent girl students who were residents of the woreda for at least 6 months were included in the study but adolescent girl students who had physical deformity were excluded due to the difficulty for anthropometric measurements. The sample size was calculated using a single population proportion formula based on the prevalence rate of underweight which was 37.8% [11] and 95% level of confidence and 5% marginal error. By adding 10% for non-respondent rate, the total sample size became 398. Lottery method was used to select fifteen schools from a total of 34 schools then the total sample size was properly allocated for the selected schools according to the number of students they have. Classes (sections) and participants were selected by the lottery method. The dependent variable was undernutrition (underweight and stunting) and the independent variables were socio-demographic factors, diet, personal hygiene and sanitation, behavior and lifestyle and anthropometric measurements. Underweight is Body Mass Index (BMI) for age < − 2 standard deviation (SD). Stunting is the height for age < − 2 SD. A pre-test was done on 5% of student adolescent girls at another woreda. Training was given for 2-days. Weighing scales were calibrated with known weight object regularly. On a daily basis, the collected information was reviewed manually. Questionnaires that had missing data were returned to the data collectors to fill the missed data by contacting the adolescent girl.

The data were analyzed using the statistical package for social sciences (SPSS) software version 20 for analysis. Descriptive statistics were done to show the magnitude of undernutrition (underweight and stunting) and participant characteristics. Associations between dependent and independent variables were checked using binary logistic regression analysis. Variables with *p* value < 0.25 in the bivariate analysis were transferred to multivariable logistic regression. In multivariable logistic regression, p-value < 0.05 was used to declare statistical significance. Adjusted odds ratio with its 95% confidence intervals was also computed.

### Results

#### Socio-demographic and economic characteristics

A total of 398 adolescent girls (10–19 years old) from fifteen schools were enrolled in this study which made the respondent rate 100%. Majority of the study groups were in the age range of 14–19 years and almost all respondents were identified as orthodox Christian. More than two-thirds of the study participants live in rural areas. The literacy rate of fathers and mothers were found to be 72.4% (284) and 51.8% (206) respectively. About 64.6% of fathers and 69.8% of mothers were identified as farmers and housewife respectively (Table 1).

**Table 1:** Socio-demographic characteristics of adolescent girls in schools of Hawzen woreda, Northern Ethiopia, 2018

Variables	Frequency (n)	Percentage (%)
Grade level		
4–8	154	38.7
9–12	244	61.3
Income		
< 300 birr	52	13.1
301–500	61	15.3
501–1000	113	28.4
> 1000	172	43.2
Family size		
≤ 4	177	44.5
> 4	221	55.5

#### Dietary factors

About 94% (374) of respondents usually ate three and more than three meals per day. Enjera with shirowet was stapled diets for 97.7% of respondents. Thirty-seven percent of the participants got their routine diet from their own product (Table 2).

**Table 2:** Diet, lifestyle, behavioral and environmental factors among adolescent girls in schools of Hawzen woreda, Northern Ethiopia, 2018

Variables	Frequency (n)	Percentage (%)
Number of meals per day		
Once	2	0.5
Twice	22	5.5
Three and above	374	94
Source of diet		
Own product	148	37.2
From market	220	55.3
From donors	30	7.5
Do you work on top of learning		
No	96	24.1
Yes	302	75.9
School distance for the home of the adolescent girl		
< 40 min	249	62.6
40–60 min	100	25.1
> 60 min	49	12.3
Do you smoke a cigarette		
No	378	95
Yes	20	5
Source of drinking water		
Tap water	384	96.5
River water	3	0.7
Protected well	7	1.8
Unprotected well	4	1
The distance of the source of water for drinking		
< 5 min	310	77.8
5–15 min	47	11.8
16–30 min	23	5.7
> 30 min	19	4.7

#### Lifestyle and behavioral factors

About 75.9% (302) of respondent adolescent girls had work other than learning. About 62.6% (249) of adolescent girls travel less than 40 min to reach the school. Ninety-five percent (378) and 77.6% (309) of the study participants neither smoke cigarettes nor drink alcohol respectively (Table 2).

#### Environmental factors

From a total of 398 respondents, about 96.5% (384) use drinking water from the improved source (pipe water). Three hundred sixty-nine participants had latrine of which 39.2% (156) of the latrine was pit latrine (Table 2).

#### The magnitude and associated factors of undernutrition among adolescent girls

About 32.2% (128) of the adolescent girls were underweight and 33.2% (133) adolescent girls were stunted. About 8.8% (35) of adolescent girls had both underweight and stunting. In the bivariate logistic regression; residence, age, father occupation, mother occupation, father education, grade level, income, meal frequency, latrine, and illness had a p-value of < 0.25 and these variables were taken to the multivariable logistic regression model. In the multivariable logistic regression; illiterate father and low-grade level were significant factors for underweight and unemployed mother was the only significant factor for stunting. Illiterate fathers were higher among underweight adolescents compared to that normal weight (AOR = 1.4, 95% CI; 1.1, 1.7). Underweight adolescents were higher among low-grade level adolescents (grade 4–8) as compared to high-grade level adolescents (9–12) (AOR = 2, 95% CI; 1.4, 2.6). Unemployed mothers were higher among stunted adolescents as compared to the counterpart (AOR = 2.2, 95% CI; 1.1, 3.3) (Table 3).

**Table 3:** Bivariate and multivariable logistic regression analysis for undernutrition among adolescent girls in Hawzen woreda, Northern Ethiopia, 2018

Variables	Underweight		COR (95% CI)	AOR (95% CI)	Stunting		COR (95% CI)	AOR (95% CI)
	Yes	No			Yes	No		
Residence								
Urban	39	114	1	1	45	108	1	1
Rural	89	156	1.7 (1.1, 2.3)	1.2 (0.3, 3.1)	88	157	1.5 (1.01, 1.9)	1.4 (0.5, 2.3)
Age								
10–13	30	36	1.9 (1.2, 2.6)	0.6 (0.2, 1)	32	34	1.6 (1.2, 2)	0.8 (0.1, 1.5)
14–19	98	234	1	1	101	231	1	1
Father occupation								
Unemployed	124	235	4.6 (1.6, 7.6)	8.1 (0.5–123.5)	122	237	1.3 (0.6, 2)	3.19 (1.93–6.4)
Employed	4	35	1	1	11	28	1	1
Mother occupation								
Unemployed	114	204	2.6 (1.02, 4.2)	2.4 (1.2, 3.6)	123	200	3.9 (1.5, 6.3)	2.2 (1.1, 3.3)*
Employed	14	66	1	1	10	65	1	1
Father education								
Illiterate	44	70	1.5 (1.02, 1.9)	1.4 (1.1, 1.7)*	92	73	5.6 (0.7, 10.5)	1.6 (1.01, 2.2)
Literate	84	200	1	1	41	192	1	1
Grade level								
4–8	66	88	2.2 (1.4, 3)	2 (1.4, 2.6)*	64	90	1.8 (1.2, 2.4)	1.5 (1.01, 1.9)
9–12	62	147	1	1	69	175	1	1
Income								
< 500 birr	13	39	0.9 (0.4, 1.4)	0.4 (0.1, 0.7)	13	39	0.7 (0.3, 1.1)	0.5 (0.1, 0.9)
500–1000 birr	23	38	1.6 (0.9, 2.3)	0.9 (0.3, 1.5)	20	41	0.9 (0.5, 1.3)	0.4 (0.02, 0.78)
1000–1500 birr	45	68	1.8 (1.1, 2.5)	1.2 (0.5, 1.9)	42	71	1.2 (0.7, 1.7)	0.8 (0.3, 1.3)
> 1500 birr	47	125	1	1	58	114	1	1
Frequency of meal per day								
1–2 meals	12	12	2.2 (1.03, 3.4)	1.06 (0.08–3.4)	9	15	1.2 (0.5, 1.9)	0.2 (0.009–4.46)
≥ 3 meals	116	258	1	1	124	250	1	1
Latrine availability								
Yes	115	254	1	1	118	251	1	1
No	13	16	0.6 (0.3, 0.9)	2.7 (0.2, 5.2)	15	14	0.4 (0.2, 0.6)	2.2 (0.6, 3.8)
Illness in 2 weeks								
Yes	31	52	1.3 (0.8, 1.8)	2.8 (1.05–4.02)	26	57	0.8 (0.5, 1.1)	2.72 (0.04–4.76)
No	97	218	1	1	107	208	1	1

1 = reference category, * = p-value < 0.05

### Discussion

This study showed that 32.2% of school adolescent girls were underweight. This magnitude is higher as compared to other studies conducted in Arsi Zone (14.5%), Ambo (27.2%), Adama city (21.3%), Bale Zone (13.7%), Arbaminch (19.7%), West Harage (24.24%), Northern Ethiopia (26.1%), Adwa town (21.4%), Addis Ababa (6.2%), Ethiopia (13.6%), Zambia (13.7%), Nigeria (18.6%), seven Africa countries (12.6%-31.9%), Latin America and Caribbean countries (3%-22%) [8, 12–24]. This might be due to the low socioeconomic status in this study area. The magnitude of underweight in this study is low as compared to the magnitude reported from studies conducted in Mekelle city (37.8%), Eastern Tigray (55%), Tigray (58.3%), Bangladesh (67%), and India (49%) [25–29]. This could be due to the time gap variation in which currently improved awareness about nutrition in adolescent parents and the current nutritional intervention. The magnitude of underweight in this study is consistent with the magnitude of underweight in Myanmar [28].

The magnitude of stunting in this study was 33.2%. This is higher than the study result from Arsi Zone (20.2%), Adama city (15.6%), West Hararge (7.2%), North Ethiopia (28.5%), Adwa town (12.2%), Eastern Tigray (25.5%), Tigray Region (26.5%), Addis Ababa (7.2%), Ethiopia (31.5%), Indonesia (23.6%) [12, 14, 17–20, 26, 27, 30]. The difference could be due to differences in socio-demographic and economic status. The magnitude of stunting in this study is lower as compared studies conducted in South East Asian countries; Bangladesh (48%), Myanmar (39%) and India (54%) [28, 29]. This difference may be due to the difference in cultural and dietary practices. The magnitude of stunting in this study is within the range of the magnitude reported from Latin America and Caribbean countries (7–43%) [24].

In this study, the father’s educational status was associated with adolescent girls underweight. This finding is in line with findings of studies conducted in Adama city, West Hararge and Ethiopia [14, 20, 27]. Higher educational status of a father can relate with a good income, good knowledge, availability and access to a balanced diet. Adolescent girls from grades four to eight were more likely to be underweight as compared to grade 9–12 adolescent girls. This finding is consistent with study findings conducted in Jimma zone and Zambia [21, 31]. Adolescent girls who attain higher grade level (9–12) can get awareness about nutrition from previous nutrition education interventions, from biology and chemistry [21]. This study showed that unemployed mothers were 2.2 times higher in stunted adolescent girls as compared their counter parts. This finding is in line with a study finding done in India [8]. This can be due to the low income, and food of unemployed mothers. The findings of this study showed that adolescent undernutrition is a public health concern which needs development and strengthening of nutrition interventions.

## Limitation

There was a possibility of recall bias and social desirability bias. Due to the nature of the study design, it was difficult to establish a cause-effect relationship.

## Data Availability

All data generated or analyzed during this study are included in this published article

## Abbreviations

AOR: Adjusted odds ratio
BMI: Body Mass Index
CI: Confidence interval
CSA: Central Statistics Agency
SD: Standard deviation
SPSS: Statistical package for social science
WHO: World Health Organization
